# Classification and Interpretation for 11 FBN1 Variants Responsible for Marfan Syndrome and Pre-implantation Genetic Testing (PGT) for Two Families Successfully Blocked Transmission of the Pathogenic Mutations

**DOI:** 10.3389/fmolb.2021.749842

**Published:** 2021-12-10

**Authors:** Songchang Chen, Hongjun Fei, Junyun Zhang, Yiyao Chen, Hefeng Huang, Daru Lu, Chenming Xu

**Affiliations:** ^1^ Obstetrics and Gynecology Hospital, Institute of Reproduction and Development, Fudan University, Shanghai, China; ^2^ The International Peace Maternity and Child Health Hospital, School of Medicine, Shanghai Jiao Tong University, Shanghai, China; ^3^ Shanghai Key Laboratory of Embryo Orignal Diseases, Shanghai, China; ^4^ State Key Laboratory of Genetic Engineering and MOE Engineering Research Center of Gene Technology, School of Life Sciences, Fudan University, Shanghai, China

**Keywords:** haplotype analysis, interpretation of sequenced variants, Marfan syndrome, pre-implantation genetic testing, molecular genetic testing, FBN1

## Abstract

**Background:** The lifespan of Marfan Syndrome (MFS) patients is shortened, especially in patients without early diagnostics, preventive treatment, and elective surgery. Clinically, MFS diagnosis is mainly dependent on phenotypes, but for children, sporadic cases, or suspicious MFS patients, molecular genetic testing, and mainly *FBN1* mutation screening, plays a significant role in the diagnosis of MFS. PGT-M gives couples that had a family history of monogenic disorders the opportunity to avoid the occurrence of MFS.

**Methods:** In this study, 11 families with MFS were recruited and complete clinical features were collected. Variants were classified and interpreted through pedigree analysis according to guidelines. Two families chose to undergo PGT-M; 16 blastocysts were biopsied and amplified. Haplotype analysis was performed to deduce the embryo’s genotype by using single nucleotide polymorphisms (SNPs) identified in each sample.

**Results:** We identified 11 potential disease-causing *FBN1* variants, six of which are novel. All variants were assessed with prediction tools to assess mutation pathogenicity, population databases to evaluate population allele frequency, literature databases to identify whether the variant had been reported in MFS patients, and multiple sequence alignment to carry out conservative analysis. Finally, nine variants were classified as likely pathogenic/pathogenic variants. Among 11 variants, eight variants were missense, and seven of them were located in the Ca-binding EGF-like motifs, moreover, half of them substituted conserved Cysteine residues. We also identified a splice site variant, a frameshift variant, and a synonymous variant. There are two variants that are *de novo* variants. PGT-M helped two MFS families give birth to a healthy baby not carrying the *FBN1* mutation.

**Conclusions**: In the present study, the *FBN1* mutation spectrum was enriched, and may help further elucidate the pathogenesis, benefiting clinical diagnosis and management of MFS. We make use of a reliable PGT-M method for the successful birth of healthy babies to two MFS families.

## Introduction

Marfan syndrome (MFS, OMIM 154700) is a connective tissue disease that is caused by mutations in fibrillin-1; it mainly affects several systems including cardiovascular, ocular, and musculoskeletal systems. MFS is inherited in an autosomal dominant pattern with an estimated prevalence of 1–5/10,000 (Ramirez and Dietz, 2007; Radke and Baumgartner, 2014). Clinically, cardiovascular manifestations (aortic dilatation and dissection) are the most serious complications that cause the death of MFS patients, although ocular and skeletal involvement may also pose a great burden on MFS patients (Ammash et al., 2008). Multiple studies confirmed that the lifespan of MFS patients is shortened, especially in patients without diagnostics and surgical treatment (Krause, 2000; Yetman et al., 2003; Groth et al., 2018).

*FBN1* gene (OMIM 134797) contained 66 exons and encoded fibrillin-1 protein; its heterozygous mutations are detected in most patients with MFS (Holcomb, 2000). Compound heterozygous or homozygous *FBN1* variants in MFS patients are rare (Arnaud et al., 2017). There are 2890 *FBN1* mutations that had been described in the professional Human Gene Mutation database. However, more pathogenic genes or atypical mutations in specific populations remain to be identified and interpreted (Arslan-Kirchner et al., 2008). Clinically, MFS diagnosis is mainly dependent on manifestations or phenotypes (Loeys et al., 2010), but for children or suspicious MFS patients, molecular genetic testing, and mainly *FBN1* mutation screening, plays a significant role in the diagnosis of MFS (Cañadas et al., 2010; Groth et al., 2017). There is a specific guideline for the interpretation of sequenced variants in the *FBN1* Gene for Marfan Syndrome besides the American College of Medical Genetics (ACMG) guideline to help us identify novel mutations in *FBN1* likely to cause MFS (Muiño-Mosquera et al., 2018). With the identification of *FBN1* as the genetic basis of MFS (Stark et al., 2020), the hope for MFS patients’ early diagnosis, preventive treatment, and elective surgery is feasible.

Pre-implantation genetic testing for monogenic diseases (PGT-M) is a part of the *in vitro* fertilization (IVF) process, which genetically profiles oocytes or embryos before implantation, and is generally available for any monogenic condition in which the causative variant is known (Besser et al., 2019; Brown, 2020; Doroftei et al., 2020). PGT-M gives couples that had a family history of monogenic disorders the opportunity to avoid the occurrence of such diseases (Hamid and Loyd, 2012; Arian et al., 2020). There are two crucial problems for couples who choose to do IVF with PGT-M: determining the disease-causing variant and effective biopsy and subsequent genetic analysis which prevent any damage to embryo viability (Priner et al., 2019).

In this study, we investigated the clinical manifestations and molecular basis of 11 unrelated suspected MFS families, to screen and identify disease-causing mutations, and two MFS families want to block the transmission of the disease by PGT-M. We reported a successful application of targeted capture sequencing and haplotype analysis-based PGT in MFS families, coupled with prenatal testing for fetal aneuploidy and large chromosomal imbalance arrangement, to help give birth to a healthy baby.

## Materials and Methods

### Study Subjects

Eleven nonconsanguineous families with MFS were recruited from International Peace Maternity and Child Health Hospital in this study. The ethics committee of International Peace Maternity and Child Health Hospital approved the project and investigators followed the principles of the Declaration of Helsinki. Informed consent was obtained from each patient and their related families before genetic testing. MFS was diagnosed according to Ghent criteria by cardiologists, ophthalmologists, internists, and geneticists.

### Clinical Data Collection

Clinical data were retrospectively collected based on patients’ medical records kept at our hospital. Case inclusion criteria and clinical data inclusion scope are (Radke and Baumgartner, 2014): diagnosed patients or family history of MFS (Ramirez and Dietz, 2007); Cardiovascular system phenotype: aortic dilatation, aortic dissection, or mitral valve prolapse (Ammash et al., 2008); Ocular system phenotype: high myopia >6.0D or ectopia lentis (Yetman et al., 2003); Skeletal system phenotype: arachnodactyly, scoliosis, pectus excavatum, or flatfeet.

### DNA Extraction and Mutation Detection

Genomic DNA was isolated from peripheral blood or using the MagNA Pure LC DNA Isolation Kit (Roche Diagnostics, GmbH, Mannheim, Germany). Whole-exome sequencing library construction and sequencing were performed using the Illumina platform by Beijing Genomics Institute (BGI) according to the manufacturer’s protocols. The detection covers exons (over 180,000) and 10bp flanking sequences of 22,000 genes. Exome sequencing was performed on the HiSeq2000 sequencing platform (Illumina). In-solution whole-exome capture and massively parallel sequencing was performed using the Agilent SureSelectXT All Exon Kit 51 Mb. Sequenced reads were collected, filtered for quality, and aligned to the human reference sequence (UCSC Genome Browser hg19) with the Burrows-Wheeler Aligner. On average, over 95% of exons were covered at >20×. Sequence variants including single-nucleotide variants (SNVs) and small insertions or deletions (indels) were annotated by ANNOVAR software. Common variants (defined as 10% frequency in 1,000 Genomes) were excluded if they were present in the dbSNP (v.142) database, the 1,000 Genomes Project, or the Exome Aggregation Consortium (ExAC) Browser. The detected variants were annotated and filtered with Annovar based on public databases [such as Mendelian Inheritance in Man (OMIM), Exome Aggregation Consortium (ExAC) Browser and MutationTaster2] in accordance with the criteria set by the American College of Medical Genetics and Genomics (ACMG)/Association for Molecular Pathology (AMP) guidelines. We focus on screen mutations of the *FBN1* (NM_000138.5) gene with bioinformatics analysis of FASTQ files. Each mutation we found will be confirmed by bidirectional Sanger sequencing.

### Familial Segregation and Classification of Variants

The sequences of screened variations sites in *FBN1* (NM_000138.5) were obtained from UCSC Human Genome Browser. Pedigree analysis was performed to identify the disease-causing mutation. The variants were detected in probands and his/her family members by polymerase chain reaction (PCR). All variants were classified according to the *FBN1*-Specific Guideline for the Interpretation of Sequenced Variants in the *FBN1* gene. The involved databases and criteria are as follows:

1) Prediction tools which are used to assess mutation pathogenicity contained SIFT (http://sift.jcvi.org), PolyPhen-2 (http://genetics.bwh.harvard.edu/pph2), Rare Exome Variant Ensemble Learner (REVEL) (https://sites.google.com/site/revelgenomics/), ClinPred (https://sites.google.com/site/clinpred/), Human Splicing Finder (http://www.umd.be/HSF3/index.html/), and NNSPLICE (http://www.fruitfly.org/seq_tools/splice.html).
2) Population databases which are used to evaluate population allele frequency information contained gnomAD (https://gnomad.broadinstitute.org) and integrated online website VarCards (http://varcards.biols.ac.cn/).
3) Literature Databases which are used to identify whether the variant had been reported in MFS patients contained Human Gene Mutation database (HGMD) (http://www.hgmd.cf.ac.uk/ac/index.php), PubMed (https://pubmed.ncbi.nlm.nih.gov), and Mastermind Genomic Search Engine (https://mastermind.genomenon.com/).
4) Some helpful databases which are used to distinguish whether the variants are located in functional domain such as Cysteine substitutions in the cb-EGF domains of fibrillin-1 are considered variants that happen in functional domains. The database contained VarSome (https://varsome.com/), subRVIS (http://subrvis.org/), and InterVar (http://wintervar.wglab.org/).

### History of 2 Marfan Syndrome Families Who Undergo PGT-M

Proband in family five is a female (III-2). She was a 26-year-old woman who was diagnosed with Marfan syndrome (MFS). Her mother and grandfather are all MFS patients and are treated for to aortic dilatation and mitral valve prolapse. She and her mother are very tall. All three MFS patients in this family suffered cardiac anomalies and skeletal dysplasia. Her father, husband, and aunt were apparently healthy. The variant is classified as uncertain significance with a pathogenic possibility of 67.5%–81.2. The patient had a strong desire to block the disease and to have an unaffected child via PGT-M. After expert consultation, informed consent from the patient and her husband, and approval from the ethics committee, the couples were determined to be pregnant through IVF and PGT-M, and signed an informed consent form for the PGT-M cycle.

Proband in family eight is a Male (I-2). He was a 36-year-old man who was diagnosed with Marfan syndrome (MFS). He had a c.5498G > T [*p*(Cys1833Phe)] mutation in *FBN1* gene. He was a typical MFS patient with disease phenotype in the Cardiovascular system, Ocular system, and Skeletal system. The variant is classified as pathogenic which is a pathogenic possibility over 99.7%. The MFS patient and his wife decided to undergo an IVF cycle associated with PGT-M and signed their informed consent.

### Preimplantation Genetic Testing and Prenatal Diagnosis

Family five and family eight obtained 18 and 10 embryos respectively; embryo biopsy was performed on Day 3 (cleavage stage) and blastocysts biopsy was performed in embryos of grade 3 or higher according to Gardner’s grading scale on day 6. The multiple displacement amplification (MDA) products and gDNA libraries were prepared and captured using a 1.5 Mb customized probe covering 350 kb upstream to 350 kb downstream of the *FBN1* gene. The SNPs identified in the couple and their parents were used for haplotype construction. Embryos diagnosed as unaffected were selected for transfer. Prenatal molecular diagnosis was performed through amniocentesis at the 13th-20th gestational week. The fetal genotype was confirmed by Sanger sequencing.

## Results

### Clinical Characteristics of MFS Probands and Patients

Clinical information of MFS probands and patients was summarized in Table 1. Affected patients from these families exhibited similar clinical symptoms of MFS. All the healthy family members had no features of MFS. We recruited 11 probands with MFS and recorded the phenotype of all MFS patients. The phenotype and family history of affected patients with *FBN1* mutation coincided with MFS except in Family 4 and Family 6, the clinical manifestations of the two families are mainly ocular defects including ectopia lentis and high myopia >6.0D. The clinical feature of the proband in Family 11 is missing since the proband died before birth.

**TABLE 1:** Clinical features of fibrillin-1 (*FBN1*) mutation patients in 11 Marfan families.

Family	Individual no	Sex	Age	Height (cm)	Cardiovascular system	Ocular system	Skeletal system
1	I-2	Male	39	195	Ao Dil; MVP	EL; S	IBL; AR; P
	I-3	Male	42	183	Ao Dil; Ao Dis; MVP	NA	IBL
	II-1	Female	15	176	NA	HM	IBL; AR
	II-4	Male	12	160	NA	NA	SCO; P
2	I-1	Female	51	177	Ao Dil; Ao Dis; MVP	EL; HM	IBL; AR; SCO
	I-2	Male	53	185	Ao Dil; Ao Dis	HM	IBL; AR; SCO
	II-2	Female	29	175	Ao Dil; Ao Dis	HM	IBL; AR; SCO
	II-3	Female	31	172	Ao Dil; Ao Dis	HM	IBL; AR; SCO
3	II-1	Female	37 (die in 40)	175	Ao Dil; Ao Dis; MVP	EL; HM	IBL; AR; P
4	I-1	Male	78	170	NA	EL	NA
	II-1	Female	55	155	NA	EL; HM	NA
	II-2	Male	55	172	NA	HM	NA
	II-4	Female	50	160	NA	EL; S	NA
	III-2	Female	28	164	NA	EL; HM	NA
5	I-1	Male	70	175	Ao Dil; MVP	NA	AR; SCO; F; P
	II-2	Female	47	177	Ao Dil; MVP	NA	IBL; AR; F; P
	III-2	Female	26	178	Ao Dil; MVP	NA	IBL; AR; SCO; P
6	I-1	Female	72	170	NA	EL; HM	NA
	II-1	Female	56	164	NA	EL; HM	NA
	II-2	Male	54	180	NA	EL; HM	NA
	II-3	Male	50	180	NA	EL; HM	NA
	III-2	Female	32	170	NA	EL; HM	NA
	III-3	Female	20	171	NA	EL; HM	NA
7	I-1	Female	61	175	NA	EL; HM	IBL; AR; SCO; F
	II-1	Female	32	167	NA	EL; HM	AR; SCO; F
8	I-2	Male	36	182	Ao Dil	HM	AR; SCO; P
9	II-2	Male	34	181	Ao Dil; MVP	EL; HM	AR; P
10	I-1	Female	59	170	Ao Dil	EL; HM	IBL; AR; SCO
	II-1	Male	30	187	Ao Dil	EL; HM	IBL; AR
11	II-1	Female	0	—	uncertain due to death of patient		

### FBN1 Mutation Screening and Pedigree Analysis

**FIGURE 1:**
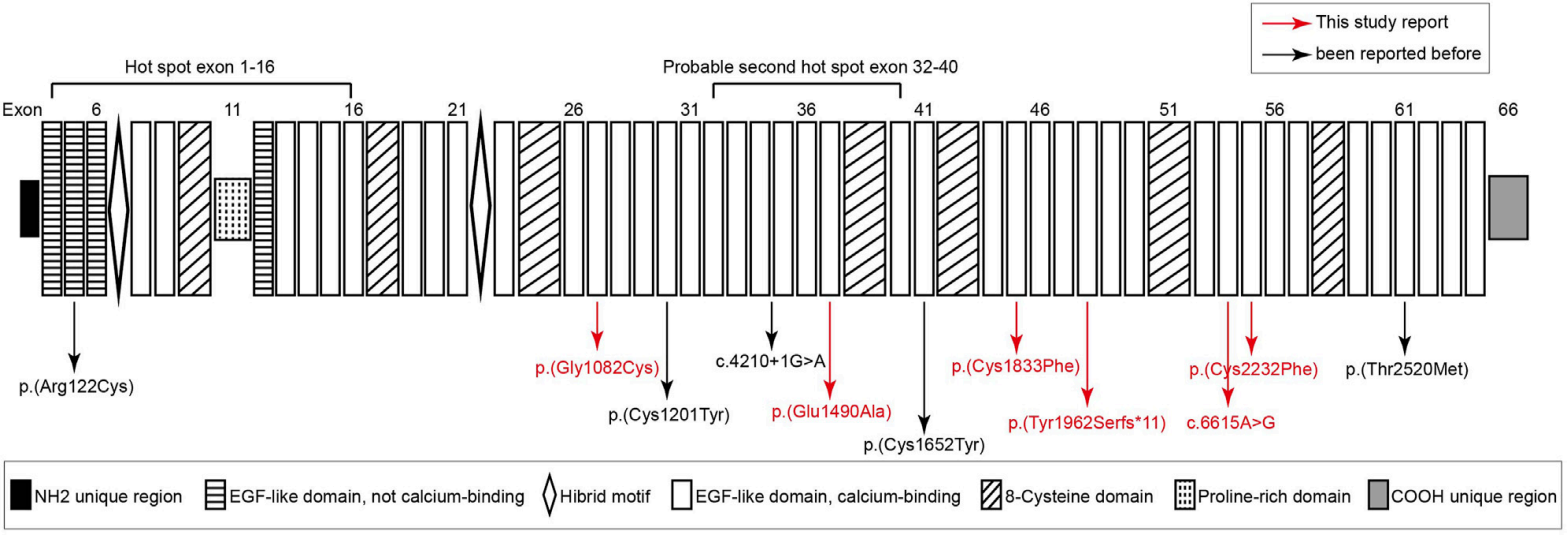
Structure diagram of *FBN1* sequence, and distribution of *FBN1* mutations identified from Marfan syndrome patients. six variants marked in red are novel variants never reported before. Except for a splicing variant (c.4210+1G > A) and c.364C > T [*p*(Arg122Cys)] that occurred in the not calcium binding EGF-like domain, other variants all occurred in the calcium binding EGF-like domain and four mutations happened in the cysteine residues of *FBN1*.

In this study, we analyzed the genomic DNA of 11 probands with MFS. The quality and reliability of targeted NGS data were evaluated based on the percentage of readable bases and the coverage depth in the targeted region, to ensure complete sequencing coverage of all coding regions in candidate genes. Altogether, 11 potentially disease-causal *FBN1* gene variants were screened out in 11 probands, 6/11 (54.5%) variants had not been reported before and 5/11 (45.5%) variants had been reported in MFS patients. We showed all mutations and their location in the protein domain of *FBN1* (Figure 1). The hot spot mutations were considered to happen frequently in exon 1–16 and secondarily exon 32–40. Among 11 variants, six variants marked in red were first reported through this study. The location and basic information of all mutations are summarized in Table 2. Except for one splicing variant, nine variants are in Ca-binding EGF-like domains and one is not in a Ca-binding EGF-like domain. There are eight missense variants, and four of eight (50%) missense variants affected conserved Cysteine residues of fibrillin 1 protein.

**TABLE 2:** *FBN1* variants identified for affected individuals in 11 Marfan families.

Family	Mutations	Exons	AA substitutions	Protein domains	Reported before or not
1	c.4955G > A	41	p.(Cys1652Tyr)	Ca-binding EGF-like motif	Ritsu Matsukawa et al., 2000
					Frank Tiecke et al., 2001
2	c.4469A > C	37	p.(Glu1490Ala)	Ca-binding EGF-like motif	This study
3	c.4210+1G > A	—	p.(?)	Intronic	Linnea M Baudhuin et al., 2015
4	c.7559C > T	61	p.(Thr2520Met)	Ca-binding EGF-like motif	Paolo Comeglio et al., 2007; Lohith Vatti et al., 2017
5	c.6615A > G	54	p.(Glu2205 = )	Ca-binding EGF-like motif	This study
6	c.3244G > T	27	p.(Gly1082Cys)	Ca-binding EGF-like motif	This study
7	c.5885_5895del	48	p.(Tyr1962Serfs*11)	Ca-binding EGF-like motif	This study
8	c.5498G > T	45	p.(Cys1833Phe)	Ca-binding EGF-like motif	This study
9	c.6695G > T	55	p.(Cys2232Phe)	Ca-binding EGF-like motif	This study
10	c.364C > T	5	p.(Arg122Cys)	Not Ca-binding EGF-like motif	Chongfei Jin et al., 2008; P Comeglio et al., 2002; Jie Li et al., 2014
					Pees C et al., 2014
11	c.3602G > A	30	p.(Cys1201Tyr)	Ca-binding EGF-like motif	Amanda Veiga-Fernández et al., 2019
					Chantal Stheneur et al., 2017
					Murat Derbenta et al., 2007

Ao Dil, aortic dilatation; Ao Diss, aortic dissection; MVP, mitral valve prolapse; EL, ectopia lentis; S, strabismus; HM, high myopia >6.0D; IBL, increased body length; AR, arachnodactyly; SCO, scoliosis; P, pectus excavatum/pectus carinatum; F, flatfeet; NA, no abnormal.

**FIGURE 2:**
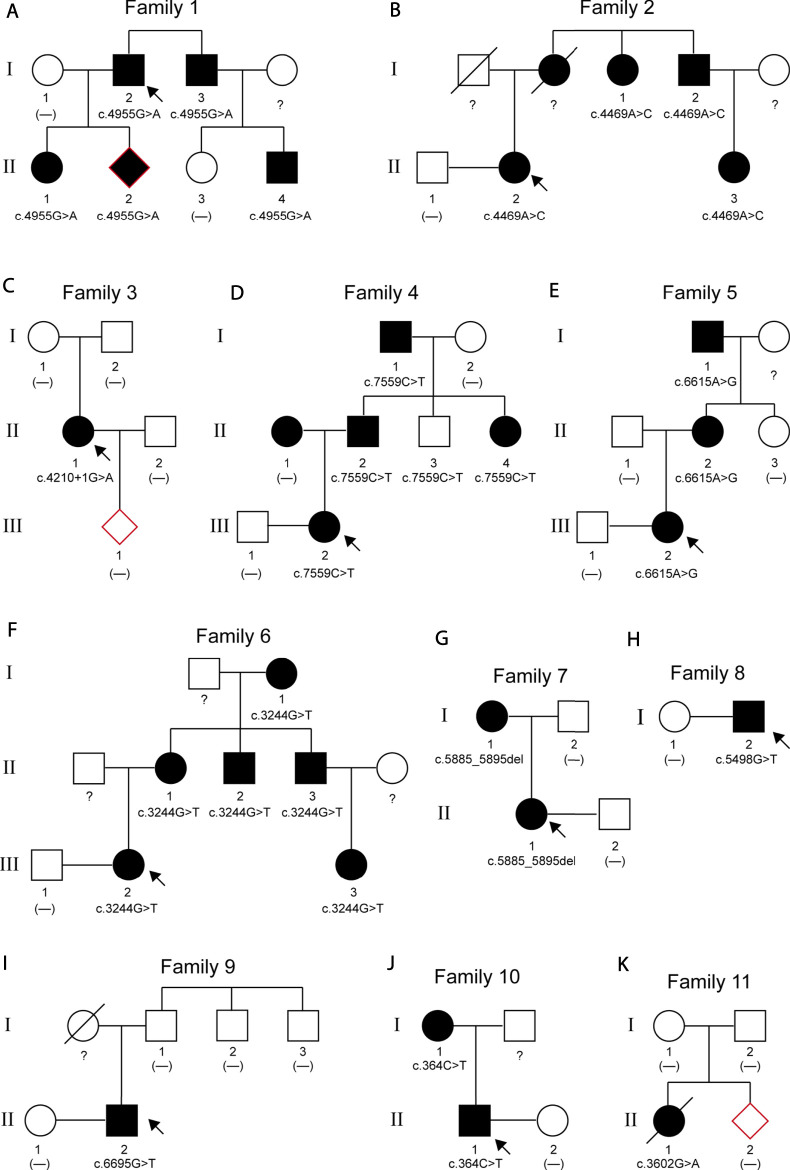
Pedigrees of 11 families with MFS. The arrows indicate the proband of each family. Squares represent males; circles represent females; solid symbols indicate affected patients; open symbols indicate unaffected subjects; a slash through the symbol means deceased.

**FIGURE 3:**
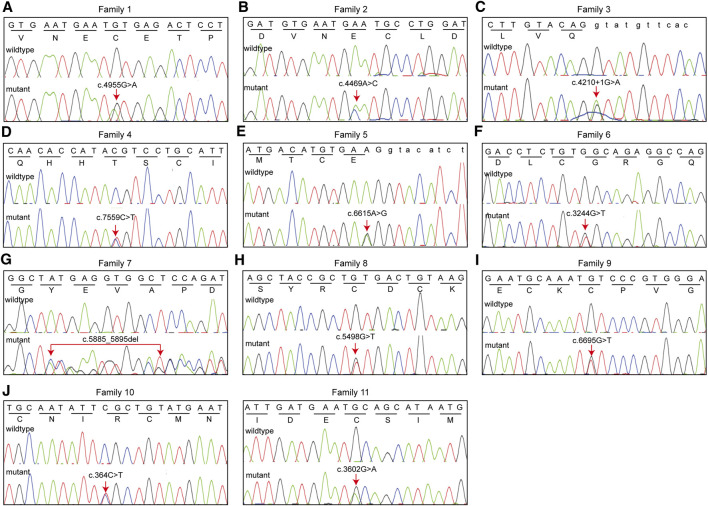
Eleven *FBN1* mutations identified in 11 families with MFS were validated using Sanger sequencing. We presented nucleotide sequence and corresponding amino acid sequence.

Pedigree analyses were performed to obtain familial segregation data and determine whether the mutation is a *de novo* mutation. Each variant, considered as a causative candidate and pathogenic mutation, was further validated using the Sanger sequencing method in other family members. Figure 2 is the pedigree chart of 11 MFS families. *FBN1* mutations in Family 3 and Family 11 were do novo mutations. Figure 3 showed the validation result of *FBN1* mutations in MFS families by sanger sequencing.

### FBN1 Variant Classification According to the ACMG Guidelines

**FIGURE 4:**
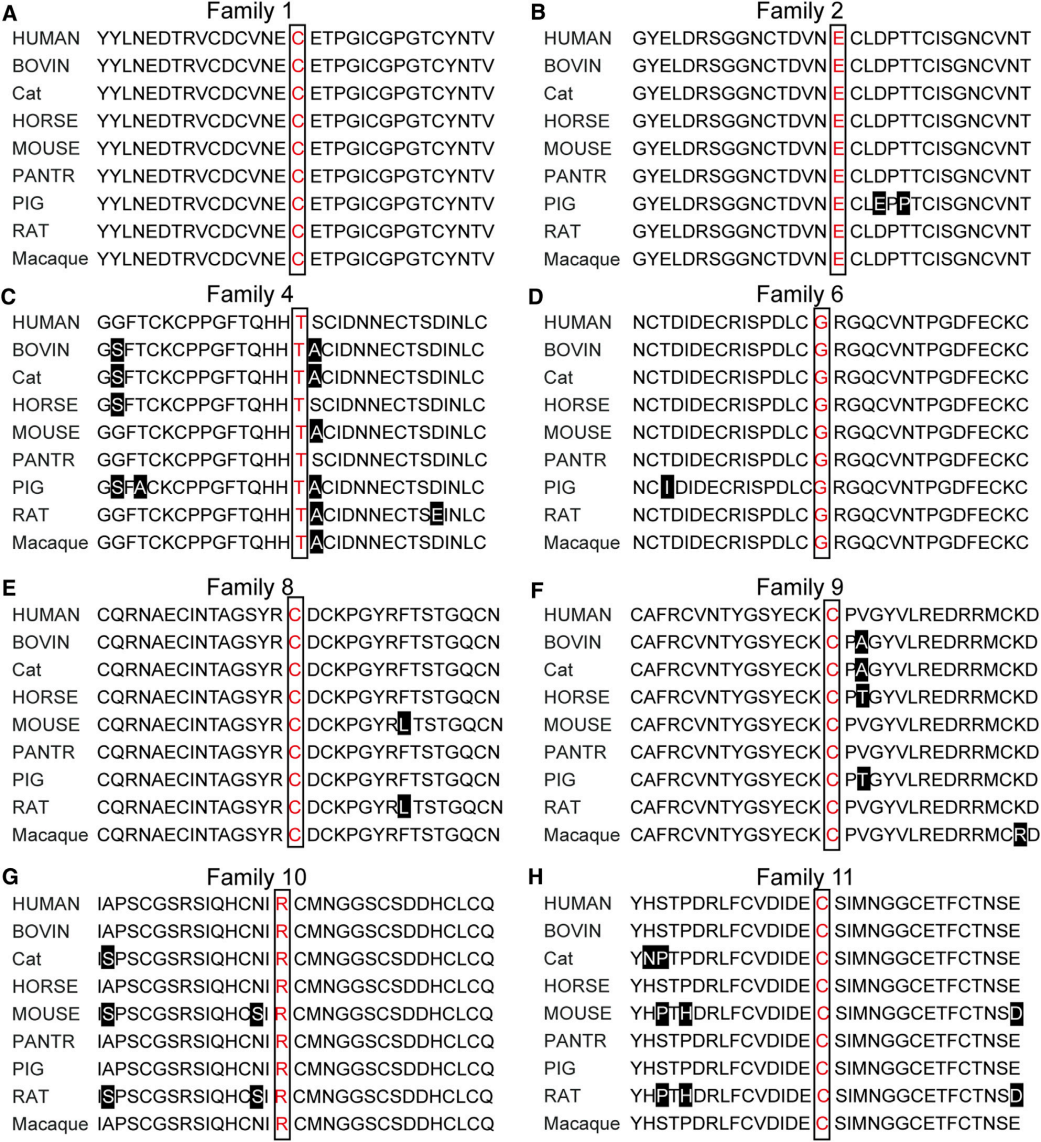
Orthologous protein sequence alignment of *FBN1* sequence. Conservation analysis among nine species showed all 11 mutations sites happened in a highly conserved region of *FBN1* among different species.

To identify disease-causing mutations for MFS families, stringent criteria according to ACMG guidelines were performed. We carefully examined all available literature and mutation-related database for sequence variant interpretation. In Table 3, allele frequencies of variants detected in populations, pieces of computational evidence of pathogenicity prediction, the clinical significance of variants in Clinvar, and whether the variant is a *de novo* variant are listed. We explored the classification evidence for each variant and did sequence variant interpretation according to the ACMG criterion. In Figure 4, conservation analysis of the related homologous proteins in eight *FBN1* missense mutation sites was conducted by referring to the UniProt database. Orthologous protein sequence alignment for eight missense mutations showed the eight mutation sites happened in a highly conserved region of *FBN1* among different species. Finally, we confirmed 11 causative candidate and pathogenic heterozygous mutations including five known mutations of the *FBN1* gene in the patients, including c.4955G > A [p(Cys1652Tyr)], c.4210+1G > A [p(?)], c.7559C > T [p(Thr2520Met)], c.364C > T [p(Arg122Cys)], and c.3602G > A [p(Cys1201Tyr)], as well as six novel mutations never reported before, including c.4469A > C [p(Glu1490Ala)], c.6615A > G [p(Glu2205 = )], c.3244G > T [p(Gly1082Cys)], c.5885_5895del [p(Tyr1962Serfs*11)], c.5498G > T [p(Cys1833Phe)], and c.6695G > T [p(Cys2232Phe)].

**TABLE 3:** Classification of pathogenicity of *FBN1* mutations identified from 11 Marfan families.

FBN1 mutations	Allele frequency	SIFT	REVEL	Polyphen2	Clinical significance in clinvar	De novo	Evidence criterion	ACMG classification
	In gnomAD							
c.4955G > A	0	Damaging (0.0)	Damaging (0.982)	Probably_Damaging (0.999)	Pathogenic (One star)	No	PM1; PM2; PS4_Moderate; PP1_Moderate; PP2; PP3; PP4	Likely pathogenic
*p*.(Cys1652Tyr)								
c.4469A > C	0	Damaging (0.0)	Damaging (0.946)	Probably_Damaging (0.998)	—	No	PM2; PM1; PM5; PP1; PP2; PP3	Likely pathogenic
*p*.(Glu1490Ala)								
c.4210+1G > A	—	—	—	—	Pathogenic/Likely pathogenic (Zero star)	Yes	PVS1; PM2; PM6; PS4_Supporting	Pathogenic
*p*.(?)								
c.7559C > T	0.00004246	Tolerable (0.086)	Damaging (0.656)	Probably_Damaging (0.927)	Uncertain significance (One star)	No	BS4; PS4_Supporting; PM2; PP2	Uncertain significance
*p*.(Thr2520Met)								
c.6615A > G	—	—	—	—	—	No	PM2; PP1; PP3; PP4	Uncertain significance
*p*.(Glu2205 = )								
c.3244G > T	0	Damaging (0.0)	Damaging (0.728)	Probably_Damaging (1.000)	—	No	PM1; PM2; PP3; PP2; PP1_Strong	Likely pathogenic
*p*.(Gly1082Cys)								
c.5885_5895del	—	—	—	—	—	No	PVS1; PM2; PP4	Pathogenic
*p*.(Tyr1962Serfs*11)								
c.5498G > T	0	Damaging (0.0)	Damaging (0.988)	Probably_Damaging (0.996)	—	Unknow	PM1; PM2; PP2; PP3; PM5_Strong; PP4	Pathogenic
*p*.(Cys1833Phe)								
c.6695G > T	0	Damaging (0.0)	Damaging (0.975)	Probably_Damaging (0.997)	—	No	PM1; PM2; PP2; PP3; PM5_Strong	Pathogenic
*p*.(Cys2232Phe)								
c.364C > T	0.000003986	Damaging (0.034)	Damaging (0.601)	Probably_Damaging (0.993)	Pathogenic (Two star)	No	PM1; PM2; PS4_Moderate; PP2; PP3; PP4; PP1_Strong	Pathogenic
*p*.(Arg122Cys)								
c.3602G > A	0	Damaging (0.0)	Damaging (0.967)	Probably_Damaging (0.986)	Likely pathogenic (Zero star)	Yes	PM1; PM2; PM6; PP2; PS4_Moderate; PP3	Likely pathogenic
*p*.(Cys1201Tyr)								

**FIGURE 5:**
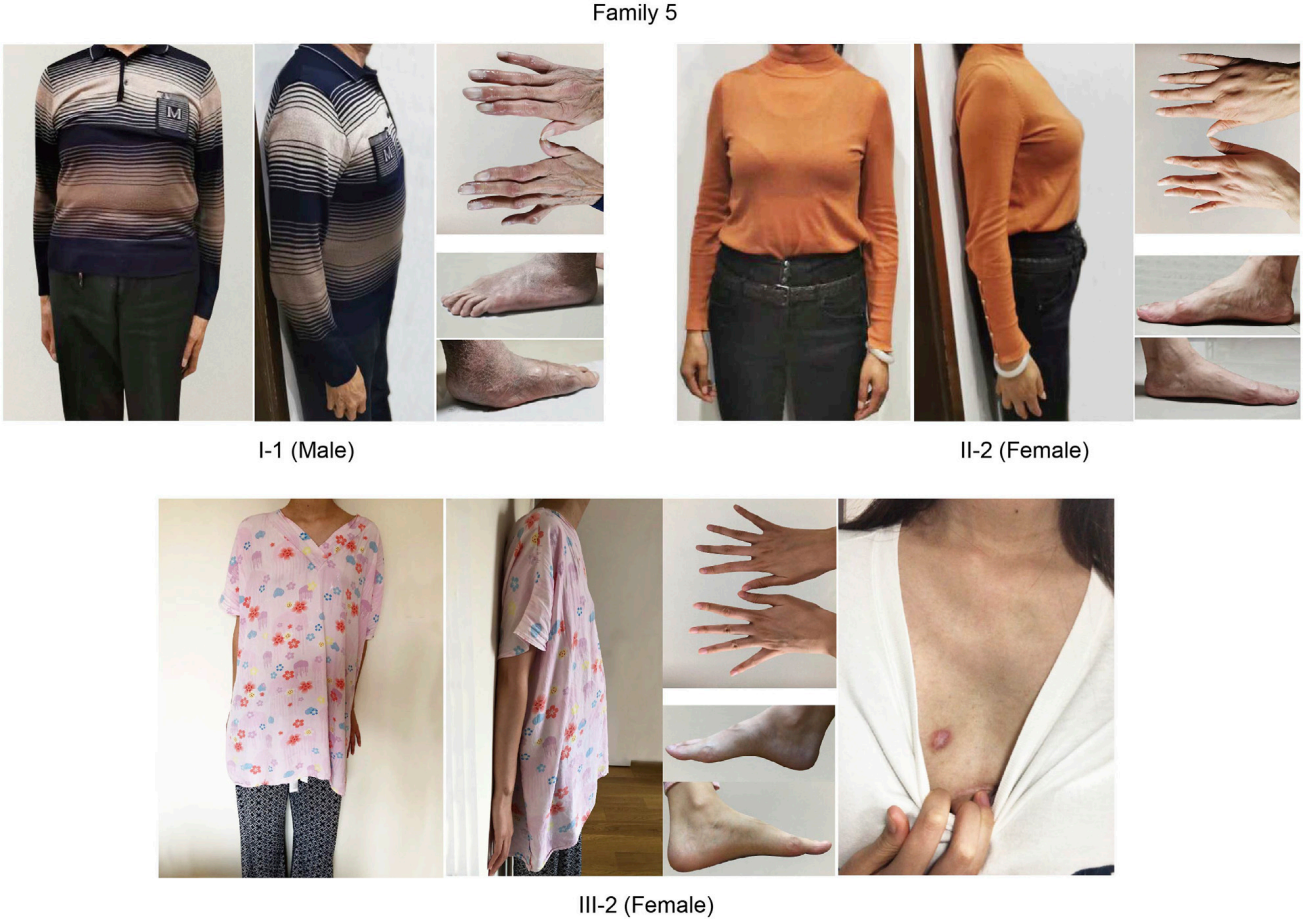
Clinical features of MFS patients in Family five who chose to do IVF with PGT-M. All three patients posted cardiac surgery. They all had a clinical manifestation of arachnodactyly and pectus excavatum/pectus carinatum. I-1 and III-2 had scoliosis. I-1 and II-2 have flatfeet.

For the *FBN1* gene, missense variants are a common mechanism of MFS disease (PP2). c.4955G > A [*p*(Cys1652Tyr)] is a missense variant (PP2), it had been reported by several groups in patients with MFS (PS4_Moderate). The variant is not present in GnomAD and ExAC (PM2). The variant results in a Cysteine substitution in the critical function domain of the calcium-binding EGF-like domain of FBN1 protein (PM1). In Family 1, the mutation was segregated with the phenotype in five affected patients and was absent in unaffected individuals (eLOD = 1.20, PP1_Moderate). Phenotype and family history of this family and reported patients were consistent with *FBN1*-induced MFS (PP4). Cys1652 is conserved between species and predicted to be disease-causing according to in silico prediction (PP3). In summary, this variation is classified as “likely pathogenic variation”. In August 2016, the patient and his wife became pregnant naturally, and the fetus (II-2) was diagnosed with *FBN1* c.4955G > A mutation after prenatal diagnosis.

c.4210+1G > A [p(?)] is in intron 34 and affects the canonical +1 donor splice site most likely leading to abnormal splicing (PVS1). It has been reported previously in association with MFS (PS4_Supporting). The mutation was not detected in the samples of proband’s parent (PM6) and was not present in GnomAD (PM2). In summary, this variation is classified as “pathogenic variation”. In December 2019, the patient and his wife became pregnant naturally, and the fetus (III-1) did not carry this mutation after prenatal diagnosis.

c.7559C > T [p(Thr2520Met)] is a missense variant (PP2), and is found at a very low frequencies in GnomAD (0.00004246) (PM2). Although the variant had been reported in an MFS patient (PS4_Supporting), in our study, the mutation was not segregated with the phenotype in five affected patients and was detected in unaffected individuals (BS4). In summary, this variation is classified as “Uncertain significance variation”.

c.364C > T [p(Arg122Cys)] is a missense variant (PP2) and located in a critical function domain of the not calcium-binding EGF-like domain of FBN1 protein (PM1). The variant is found at a very low frequency in GnomAD (0.000003986) (PM2) and many pieces of prediction software predict this variant to be damaging (PolyPhen2, SIFT, REVEL) (PP3). The variant had been reported in MFS patients before (PS4_Moderate) and was segregated with the phenotype in eight affected patients (eLOD = 2.00, PP1_Strong). In Family 10 and the previous reports, phenotypes and family histories of patients were consistent with MFS (PP4). In summary, this variation is classified as “pathogenic variation”.

c.3602G > A [p(Cys1201Tyr)] is a missense variant (PP2) and results in a Cysteine substitution in the critical function domain of the calcium-binding EGF-like domain of FBN1 protein (PM1). The mutation was not present in GnomAD (PM2) and was predicted to be disease-causing by in silico analysis (PP3). In Family 11, the mutation is a *de novo* mutation (PM6), and the mutation had been reported in the literature (PS4_Moderate). In summary, this variation is classified as “likely pathogenic variation”. In May 2019, the parents of the probands conceived naturally, and the fetus (II-2) did not carry *FBN1* c.3602G > A mutation after prenatal diagnosis.

c.4469A > C [p(Glu1490Ala)] is a novel missense variant (PP2) and located in a critical function domain of the calcium-binding EGF-like domain of FBN1 protein (PM1). The mutation was not present in GnomAD (PM2) and was predicted to be disease-causing by in silico analysis (PP3). In Family 2, the mutation was segregated with the phenotype in four affected patients and was absent in unaffected individuals (eLOD = 0.90, PP1). Moreover, mutation at Glu1490 locus [p(Glu1490Lys)] had been classified as likely pathogenic (PM5). In summary, this variation is classified as “likely pathogenic variation".

c.6615A > G [p(Glu2205 = )] is a novel synonymous variant that had not been reported before, all three MFS patients had c.6615A > G mutation in *FBN1* but the other healthy members in her family did not carry this mutation (eLOD = 0.90, PP1). The variant is absent from all population databases (PM2), and multiple lines of computational evidence support a deleterious effect on the gene product through disturb splicing (PP3). The phenotype and family history of the proband in this family are highly consistent with MFS characteristics (PP4). All three MFS patients in this family suffered cardiac anomalies and skeletal dysplasia. In Figure 5, we can see three affected patients have arachnodactyly, flatfeet, pectus excavatum, or pectus carinatum. In summary, this variation is classified as “Uncertain significance variation”. The variant is classified as uncertain significance with a pathogenic possibility of 67.5%–81.2. The patient had a strong desire to block the disease and to have an unaffected child via PGT-M. After expert consultation, informed consent from the patient and her husband, and approval from the ethics committee, the couples were determined to be pregnant through IVF and PGT-M, and signed an informed consent form for the PGT-M cycle.

c.3244G > T [*p*(Gly1082Cys)] is a novel missense variant (PP2) and located in a critical function domain of the calcium-binding EGF-like domain of FBN1 protein (PM1). The mutation was not present in GnomAD (PM2) and was predicted to be disease-causing by in silico analysis (PP3). In Family 6, the mutation was segregated with the phenotype in six affected patients and was absent in unaffected individuals (eLOD = 1.50, PP1_Strong). In summary, this variation is classified as “likely pathogenic variation”.

c.5885_5895del [*p*(Tyr1962Serfs*11)] is a novel frameshift variant that likely leads to a truncated protein (PVS1). The variant is absent from all population databases (PM2), and the phenotype of affected patients is consistent with MFS (PP4). In summary, this variation is classified as “pathogenic variation”.

c.5498G > T [*p*(Cys1833Phe)] and c.6695G > T [*p*(Cys2232Phe)] are all novel missense variants (PP2) and result in a Cysteine substitution in the critical function domain of the calcium-binding EGF-like domain of FBN1 protein (PM1). The mutations were not all present in GnomAD (PM2) and were predicted to be disease-causing by in silico analysis (PP3). In Family eight and Family 9, the phenotype of affected patients is consistent with MFS (PP4). Moreover, mutation at Cys1833 locus [*p*(Cys1833Arg), *p*(Cys1833Ser)], and mutation at Cys2232 locus [*p*(Cys2232Tyr), *p*(Cys2232Arg)] had been reported to be pathogenic (PM5_Strong). In summary, the two variations are classified as “pathogenic variation".

### Haplotype Analysis and PGT-M Cycles

**FIGURE 6:**
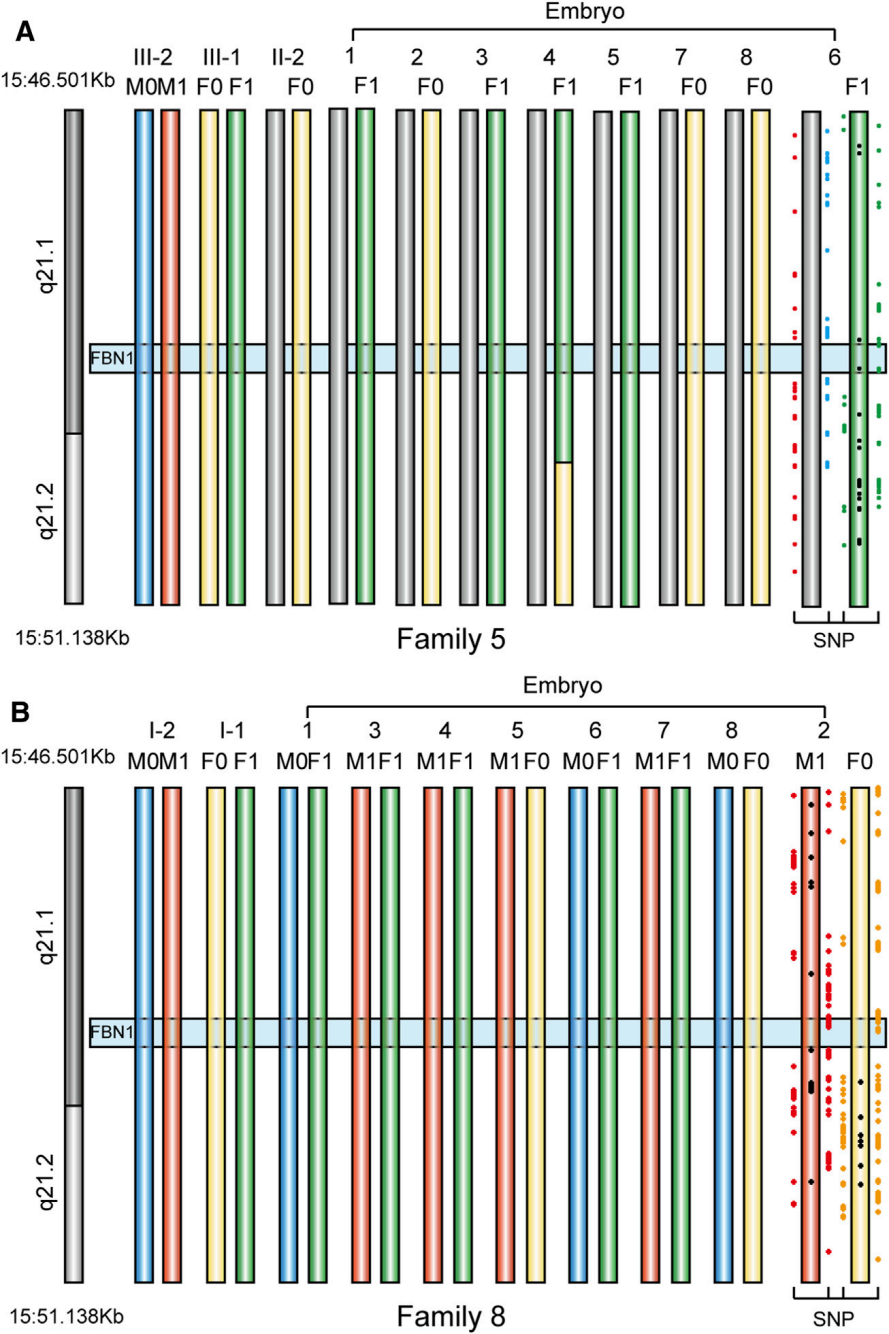
The haplotype in *FBN1* gene in eight embryos from two families. We showed informative SNPs that supported the haplotype of only one embryo used for implantation **(A)** PGT haplotype analysis in embryos 1 to eight in Family 5. F0 means Female disease-causing chromosome, F1 means Female normal chromosome, M0 and M1 means Male normal chromosome. Embryo 4 had a recombination event in the maternal allele (length 869.56 Kb), but it did not influence the genotype deduction of the *FBN1* gene, because the recombination loci were outside the gene region **(B)** PGT haplotype analysis in embryos 1 to eight in Family 8. M0 means Male disease-causing chromosome, M1 means Male normal chromosome, F0 and F1 mean Female normal chromosome.

In Family 5, we defined the haplotype linked to c.6615A > G and associated with MFS as F0, and the haplotype linked to wildtype allele as F1. In Family 8, we defined the haplotype linked to c.5498G > T and associated with MFS as M0, while the haplotype linked to wildtype allele was defined as M1. The genotypes of 16 embryos were all successfully determined using the Hidden Markov model approach (Figure 6; Table 4). In Family 5, five embryos were free of maternal *FBN1* c.6615A > G variant; embryos 2, 7, and 8 were carriers of c.6615A > G variant. In Family 8, five embryos were free of paternal *FBN1* c.5498G > T variant; embryos 1, 6, and 8 were carriers of c.5498G > T variant.

**TABLE 4:** Details of haplotype in *FBN1* gene in embryos from two Marfan families who chose to do IVF with PGT-M.

Family number	Name	Haplotypes	Genotypes	PGT-M results	Numbers of informative SNPs supported each haplotype			
					F0	F1	M0	M1
Family 5	Embryo 1	F1/	N	normal	0	35	—	—
	Embryo 2	F0/	c.6615A > G	heterozygosis	32	0	—	—
	Embryo 3	F1/	N	normal	0	28	—	—
	Embryo 4	F1/	N	normal	1	26	—	—
	Embryo 5	F1/	N	normal	1	23	—	—
	Embryo 6	F1/	N	normal	0	30	—	—
	Embryo 7	F0/	c.6615A > G	heterozygosis	31	2	—	—
	Embryo 8	F0/	c.6615A > G	heterozygosis	35	0	—	—
Family 8	Embryo 1	M0/F1	c.5498G > T	heterozygosis	2	62	60	2
	Embryo 2	M1/F0	N	normal	67	0	0	78
	Embryo 3	M1/F1	N	normal	0	72	0	80
	Embryo 4	M1/F1	N	normal	0	100	0	83
	Embryo 5	M1/F0	N	normal	65	1	1	74
	Embryo 6	M0/F1	c.5498G > T	heterozygosis	0	66	70	0
	Embryo 7	M1/F1	N	Normal	0	57	4	71
	Embryo 8	M0/F0	c.5498G > T	heterozygosis	to construct haplotype			

Family 5: F0, Female disease-causing chromosome; F1, female normal chromosome; M0, M1, Male normal chromosome.

Family 8: M0:, Male disease-causing chromosome; M1, Male normal chromosome.

F0, F1, female normal chromosome.

### Prenatal Diagnosis

Embryo 6 in Family 5 and embryo 2 in Family 8 were selected for transfer and a successful pregnancy was confirmed by human chorionic gonadotropin (hCG) and ultrasound examination. Sanger sequencing results showed that the fetus did not carry mutations. This confirmed the accuracy of PGT-M. The chromosome imbalance anomaly results showed that no copy number variant (CNV) larger than 100 kb was identified in the fetus.

## Discussion

In this study, we collected clinical features of all MFS patients in 11 families with the monogenetic disease. We found that the clinical phenotypes of MFS patients in the same family are broadly similar and remain different. It is consistent with the reports in the literatures (Ramirez, 1996; Seo et al., 2018). In Family 1, two children (12 and 15 years old) did not have cardiac phenotypes such as aortic dilatation, aortic dissection, and mitral valve prolapse but adults had; it reminded us that molecular genetic testing for children or atypical MFS patients is important and to warn patients to take medical prophylaxis to prevent cardiovascular pathologies causing the highest mortality in MFS (Comeglio et al., 2007; Franken et al., 2017; Manchola-Linero et al., 2018).

Here, we identified 11 potential disease-causing *FBN1* variants in patients with Marfan syndrome, and six of them are novel. Among 11 variants, eight variants were missense, and seven of them were located in the Ca-binding EGF-like motifs, moreover half of them substituted conserved Cysteine residues. According to previous reports, Cysteine residues are highly conserved in fibrillin 1 (Corson et al., 2004), mutations affecting Cysteine residues may disrupt one of the three disulfide bonds and critical functional domain (Schrijver et al., 1999; Faivre et al., 2007). Our results proved that Cysteine substitutions in Ca-binding EGF-like domains of fibrillin 1 may be the most common and critical causal for *FBN1* induced MFS.

Besides eight missense variants, we also identified a splice site variant, a frameshift variant, and a synonymous variant. C.6615A > G [p(Glu2205 = )] variant had never been reported before, the affected patient who carries this variant is a typical patient, her mother and grandfather all had typical MFS phenotype (pieces of variant classification evidence including: PM2, PP1, PP3 and PP4). All of them underwent cardiac surgery. The proband and her husband hope to give birth to a healthy baby. Many pieces of prediction software predicted the variant will affect mRNA splicing, however, more functional evidence is needed.

According to statistics, over 25% of MFS patients are sporadic cases, and these patients always have the severe neonatal phenotype (Collod-Béroud and Boileau, 2002; Tan et al., 2017; Weerakkody et al., 2018). In our study, II-1in Family 3 and II-1 in Family 11 are sporadic cases, their parents are normal, they had no family history, and the variants are *de novo*. Obviously, they had a severe disease phenotype, II-1in Family 3 died when she was 40 years old and II-1 in Family 11 died after birth. The clinical criteria in the revised Ghent nosology are not always suitable for children, particularly those sporadic cases. It further proved that molecular diagnosis of MFS is useful and necessary (Toudjarska et al., 2001; Ades and Group, 2007). Further, making the pathogenesis clear can help patients block the transmission of the pathogenic *FBN1* mutation by PGT and produce healthy babies.

Due to phenotypic variability, a high rate of sporadic cases, and lack of a genetic or biochemical test for MFS, the actual incidence of MFS may be considerably higher than the reported 1–5/10,000 (Chiu et al., 2014). And clinical demand for PGT has increased as research and cases on MFS increase. Over the past few decades, PGT always used STR as a genetic marker, but it is time-consuming to select appropriate markers. The capture sequencing and linkage analysis of SNPs located nearby the gene of interest provides a convenient and efficient way for PGT-M experiment design (Brezina et al., 2016). In two families that underwent PGT-M, informative SNPs were distributed from upstream to downstream of the *FBN1* gene, ensuring any recombination will be identified. We determined each embryo successfully. Embryo 4 in Family five had a recombination event in the maternal allele (869.56 Kb), but it did not influence the genotype deduction of the *FBN1* gene, because the recombination loci were outside the gene region. Of course, we did invasive prenatal diagnosis for two families who underwent PGT-M to avoid misdiagnosis (Chen et al., 2018).

## Conclusion

We identified six novel variants and five known variants in the *FBN1* gene from 11 Chinese families with MFS. We performed detailed classification and interpretation for 11 variants. In the present study, the *FBN1* mutation spectrum was enriched and may help further elucidate the pathogenesis, benefiting clinical diagnosis and management of MFS. We make use of a reliable PGT-M method to enable the successful birth of healthy babies for two MFS families.

## Abbreviations

ACMG, American College of Medical Genetics and Genomics; IVF, *in vitro* fertilization; MDA, multiple displacement amplification; MFS, Marfan syndrome; PGT, Pre-implantation genetic testing; PGT-M, Pre-implantation genetic testing for monogenic diseases

## Data Availability

The data presented in the study are deposited in the Sequence Read Archive (SRA) repository, accession number PRJNA778868.

## Abbreviations

ACMG, American College of Medical Genetics and Genomics; IVF, in vitro fertilization; MDA, multiple displacement amplification; MFS, Marfan syndrome; PGT, Pre-implantation genetic testing; PGT-M, Pre-implantation genetic testing for monogenic diseases.
